# The V1-V3 region of a brain-derived HIV-1 envelope glycoprotein determines macrophage tropism, low CD4 dependence, increased fusogenicity and altered sensitivity to entry inhibitors

**DOI:** 10.1186/1742-4690-5-89

**Published:** 2008-10-06

**Authors:** Fiorella Rossi, Bianca Querido, Manideepthi Nimmagadda, Simon Cocklin, Sonia Navas-Martín, Julio Martín-García

**Affiliations:** 1 Department of Microbiology and Immunology and Center for Molecular Virology and Neuroimmunology, Institute of Molecular Medicine and Infectious Disease, Drexel University College of Medicine, Philadelphia, PA 19102, USA; 2 Department of Biochemistry and Molecular Biology, Drexel University College of Medicine, Philadelphia, PA 19102, USA

## Abstract

**Background:**

HIV-1 infects macrophages and microglia in the brain and can cause neurological disorders in infected patients. We and others have shown that brain-derived envelope glycoproteins (Env) have lower CD4 dependence and higher avidity for CD4 than those from peripheral isolates, and we have also observed increased fusogenicity and reduced sensitivity to the fusion inhibitor T-1249. Due to the genetic differences between brain and spleen *env *from one individual throughout gp120 and in gp41's heptad repeat 2 (HR2), we investigated the viral determinants for the phenotypic differences by performing functional studies with chimeric and mutant Env.

**Results:**

Chimeric Env showed that the V1/V2-C2-V3 region in brain's gp120 determines the low CD4 dependence and high avidity for CD4, as well as macrophage tropism and reduced sensitivity to the small molecule BMS-378806. Changes in brain gp41's HR2 region did not contribute to the increased fusogenicity or to the reduced sensitivity to T-1249, since a T-1249-based peptide containing residues found in brain's but not in spleen's HR2 had similar potency than T-1249 and interacted similarly with an immobilized heptad repeat 1-derived peptide in surface plasmon resonance analysis. However, the increased fusogenicity and reduced T-1249 sensitivity of brain and certain chimeric Env mostly correlated with the low CD4 dependence and high avidity for CD4 determined by brain's V1-V3 region. Remarkably, most but not all of these low CD4-dependent, macrophage tropic envelopes glycoproteins also had increased sensitivity to the novel allosteric entry inhibitor HNG-105. The gp120's C2 region asparagine 283 (N283) has been previously associated with macrophage tropism, brain infection, lower CD4 dependence and higher CD4 affinity. Therefore, we introduced the N283T mutation into an *env *clone from a brain-derived isolate and into a brain tissue-derived *env *clone, and the T283N change into a spleen-derived *env *from the same individual; however, we found that their phenotypes were not affected.

**Conclusion:**

We have identified that the V1-V3 region of a brain-derived envelope glycoprotein seems to play a crucial role in determining not only the low CD4 dependence and increased macrophage tropism, but also the augmented fusogenicity and reduced sensitivity to T-1249 and BMS-378806. By contrast, increased sensitivity to HNG-105 mostly correlated with low CD4 dependence and macrophage tropism but was not determined by the presence of the brain's V1-V3 region, confirming that viral determinants of phenotypic changes in brain-derived envelope glycoproteins are likely complex and context-dependent.

## Background

Human immunodeficiency virus type 1 (HIV-1) envelope glycoproteins (Env), the heavily glycosylated surface gp120 and the non-covalently associated transmembrane subunit gp41, are organized on the virion surface as trimeric spikes and mediate viral entry into susceptible cells. The surface gp120 is composed of a core of conserved regions (C1-C5), shielded by variable loop regions (V1-V5) formed by disulfide bonds (except V5) that retain a large degree of flexibility. The gp41 ectodomain (gp41e) contains the fusion peptide, which is inserted into the membrane of the target cells, as well as two heptad repeat (HR) domains (amino-terminal or HR1 and carboxy-terminal or HR2) that are involved in the formation of a fusion intermediate, the six-helix bundle, through conformational rearrangements following receptor interaction. HIV-1 infection requires two sequential and specific binding steps: first, to the CD4 antigen present in CD4^+ ^T-cells, monocyte/macrophages and other cells; and second, to a member of the chemokine receptor subfamily, within the G protein-coupled, seven-transmembrane domain family of receptors, mainly CCR5 and/or CXCR4.

Structural analysis of unliganded gp120 from the related simian immunodeficiency virus has suggested that the large gp120 region involved in binding to CD4, the CD4-binding site (CD4bs), may only form a stable, binding-competent conformation when gp120 actually engages CD4 [1]. The interaction with CD4 triggers a rather large conformational change in gp120 that results in the formation and/or exposure of highly conserved regions previously folded into the core structure and/or sheltered by the variable loops and the glycans covering the outer domain of gp120 [2-9]. These CD4-induced regions contain discontinuous structures that react with certain human neutralizing monoclonal antibodies (mAbs) (e.g., 17b), which inhibit chemokine receptor binding to gp120 [2,5,7-15], and therefore constitute a high-affinity binding site for the co-receptor molecule. Chemokine receptor binding by gp120 has been suggested to occur first through the amino terminus, which then allows interaction with the second extracellular loop, and subsequently triggers further conformational changes on gp120 that are transduced to gp41 and lead to the fusion-active conformation of HIV-1 Env [16-21] and the formation of a fusion pore.

HIV-1 infection of the central nervous system (CNS) seems to occur early after primary infection. Subsequently, HIV-1-infected individuals may develop a neurological syndrome ranging from the mild minor cognitive/motor disorder to HIV-associated dementia, although significant neurological dysfunction and neurodegeneration are typical in advanced stages of disease [22]. Although anti-retroviral therapy has decreased the incidence of HIV-associated dementia, neurological abnormalities continue to be a relevant problem among all HIV-positive individuals [22,23]. HIV-1 likely enters the CNS as cargo in virus-infected monocytes migrating into the brain to replenish the population of perivascular macrophages. Accordingly, perivascular macrophages and microglia (long-lived, brain resident macrophages) seem to be responsible for most of the viral production within the brain. Multinucleated giant cells, the end product of fusion between infected and uninfected cells, are the principal neuropathological finding of HIV-1 infection in the brain and hallmark of HIV-1 encephalitis [24-27].

Microglia and perivascular macrophages may survive for long time after infection. They express CCR5, CXCR4 and some minor co-receptors [28], but similarly to macrophages from other tissues, CD4 expression is either undetectable or very low [29-32]. Hence, the nature of the infected cells and the special immunological status of the brain may allow viral persistence and replication in the brain. Several studies have described that viruses isolated from the brain display macrophage tropism and mainly use CCR5 to infect microglia [33-38], suggesting that viral tropism for microglia and macrophages may be determined by similar mechanisms [39-41]. In addition, numerous studies support that HIV-1 evolve independently within the CNS and in particular the brain [42-48] probably leading to compartmentalization and a progressive adaptation to this niche.

We previously described that a primary peripheral isolate adapted *in vitro *to grow in pure human adult microglial cultures developed increased fusogenicity and an improved ability to use low levels of CD4 on the target cell membrane for fusion and infection (low CD4 dependence) [49-51], which correlated with higher avidity and affinity in the interaction with CD4 [52]. The phenotypic changes mapped to the envelope glycoproteins, which differed only in 8 amino acids, and specifically to four amino acid changes in the V1/V2 region of gp120, which probably result in a more open or partially-triggered gp120 conformation. Subsequently, we hypothesized that HIV-1 could adapt within the brain compartment to specifically improve its ability to use low levels of CD4 for entry/infection on target cells, and therefore viruses with low CD4 dependence and high CD4 affinity might be naturally selected *in vivo *in the CNS of HIV-1-infected individuals.

We and others have investigated whether viruses or Env with these particular phenotypes are present among primary isolates in the brain/CNS of HIV-1-infected individuals [35,44,53-57]. In this sense, Peters et al. have reported that the macrophage tropism of brain-derived Env seems to correlate with reduced sensitivity to inhibition by an anti-CD4 mAb but not by CCR5 antagonists or gp41-targeting antibodies or fusion inhibitor peptides [55,56]. Thomas et al. have also recently reported that efficient entry into macrophages is more frequent among Env from brain than from lymphoid tissues and seems to correlate with both low CD4 dependence and overall efficiency of fusion [57]. In addition, Dunfee et al. found that asparagine in position 283 (N283) in gp120's C2 region seems to be more common in Env from brain than from lymphoid tissues, associates with the presence of dementia and could contribute to the lower CD4 dependence, slightly increased affinity for CD4 and enhanced macrophage tropism [53]. Likewise, we have previously reported that Env derived from the brain of an individual with HIV-1 encephalitis have lower CD4 dependence and higher avidity for CD4, mediate increased fusion and have significantly lower sensitivity to the fusion inhibitor T-1249, than spleen-derived Env from the same subject [54]. These envelope glycoproteins differed in numerous amino acid residues throughout gp120, with many changes clustering in or around the variable regions V1/V2 and V3, and in the HR2 region of gp41e, but not in HR1.

Several early studies identified the V3 region in gp120 as a primary determinant of macrophage tropism in envelope glycoproteins from CCR5-using peripheral isolates [58-63], although other gp120 regions, and especially the V1/V2 variable loop, have also been shown to influence the ability of viral isolates to infect macrophages [61,63-67]. Therefore, in this study we investigated the viral determinants for the phenotypic differences observed between the brain- and spleen-derived Env reported above [54], through the generation of chimeric and mutant Env and their characterization using cell-to-cell fusion and pseudotype infection assays. We found that chimeric Env containing the V1-V3 region of the brain-derived gp120 not only displayed increased macrophage tropism but also had significantly lower CD4 dependence, higher fusogenicity and lower sensitivity to the T-1249 fusion inhibitor and BMS-378806. Interestingly, most low CD4-dependent, macrophage-tropic Env also showed increased sensitivity to a novel allosteric entry inhibitor, the short peptide HNG-105. In addition, we found similar phenotypes with the Env from a brain-derived isolate and obtained evidence that the role that specific amino acid residues in gp120 play on these phenotypes is highly dependent on the Env background.

## Materials and methods

### Cells

Human embryonic kidney 293T cells and quail fibroblasts QT6 cells were cultured in Dulbecco's modified Eagle's medium (DMEM; MediaTech, Hendon, VA) supplemented with 10% heat-inactivated fetal bovine serum (FBS; Clontech, Mountain View, CA) and antibiotics. Human osteosarcoma cells (HOS) stably expressing human CD4 and CCR5 molecules (obtained through the AIDS Research and Reference Reagent Program, Division of AIDS, NIAID, NIH [ARRRP], from Dr. Nathaniel Landau) [68,69] were maintained in DMEM supplemented with 10% FBS, puromycin (1 μg/ml), mycophenolic acid (40 μg/ml), xanthine (250 μg/ml) and hypoxanthine (13.5 μg/ml). Human astroglioma U87 cells stably transfected for the expression of human CD4 and CXCR4 (obtained through the ARRRP from Drs. HongKui Deng and Dan Littman) [70] were cultured in DMEM supplemented with 10% FBS, puromycin (1 μg/ml) and G418 (300 μg/ml) (Invitrogen-Gibco, Carlsbad, CA).

Normal human monocytes were purchased as single cell suspension from the Human Immunology Core of the University of Pennsylvania Cancer Center, counted and plated for differentiation into macrophages by culturing at a density of 2.0 × 10^4 ^cells/well in 96-well plates for 7–10 days in DMEM supplemented with 5% FBS and 5% giant cell tumor conditioned media (BioVeris, Gaithersburg, MD), as previously described [71].

### Construction of chimeric and mutant envelope glycoproteins

**Figure 1:**
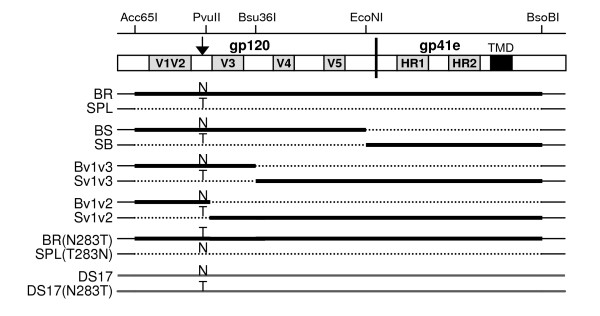
**Schematic representation of chimeric and mutant Env**. Chimeric Env constructs between brain- and spleen-derived Env of one individual were made to map the viral determinants of phenotypic changes. Single mutants were constructed to test for the role of N283 and T283 on the phenotypes observed.

The generation and characterization of full-length, functional *env *genes derived from brain (BR) and spleen (SPL) autopsy tissues of an HIV-1-infected individual with encephalitis and HIV-associated dementia (H0002GH) was previously described [54]. Subsequently, *Xho*I (in multiple cloning site of pcDNA3.1)-*EcoN*I fragments containing most of the gp120 coding regions from BR and SPL Env expression vectors were exchanged to construct the BS and SB chimeric Env, which express brain-derived gp120 with the spleen gp41 and the spleen gp120 with the brain gp41, respectively (Figure 1). Similar to the parent Env, these chimeras were functional in cell-to-cell fusion assays but did not efficiently mediate production of Env-pseudotyped viruses. Therefore, in order to achieve successful production of pseudotype viruses for infection experiments, we utilized the pHXB2-env vector (obtained through the ARRRP from Drs. Kathleen Page and Dan Littman) [72] in which highly efficient Env expression is obtained from an SV40 promoter, and *Acc65*I-*BsoB*I fragments containing a majority of the gp160 region (except the first 11 amino acids in gp120 after the signal peptide and the last 132 amino acids in gp41, all in the cytoplasmic tail and identical between the BR and SPL Env) from the BR, SPL, BS and SB Env expression vectors in pcDNA3.1 were sub-cloned into the pHXB2-env digested with the same restriction endonucleases to generate pSV-BR, -SPL, -BS, and -SB. All constructs were tested with a set of restriction endonucleases that either cut or do not cut the brain and spleen-derived gp120 and gp41 to confirm the identity of the gp120 and gp41 expressed from each construct. Furthermore, chimeric Env containing the brain V1/V2-C2-V3 or only the V1/V2 gp120 fragments in the context of the spleen Env (named Bv1v3 and Bv1v2, respectively) and the spleen V1/V2-C2-V3 or only the V1/V2 gp120 fragments in the context of the brain Env (named Sv1v3 and Sv1v2, respectively) were constructed by exchanging *Xho*I-*Bsu36*I and *Xho*I-*Pvu*II fragments of BR and SPL parental Env in pcDNA3.1, and these were subsequently sub-cloned into the corresponding pSV-BR or pSV-SPL using *Acc65*I and *BsoB*I to generate pSV-Bv1v3, pSV-Bv1v2, pSV-Sv1v3 and pSV-Sv1v2 (Figure 1).

To evaluate the role of N283 in various Env backgrounds, the Env mutants pSV-BR(N283T) and pSV-SPL(T283N) where the N283 present in the brain Env was mutated to T and the T283 present in the spleen Env was mutated to N, respectively, were created using QuikChange site-directed mutagenesis (Stratagene, La Jolla, CA) following manufacturer's instructions. The presence of the intended mutations was verified by sequence analysis.

Finally, we also characterized several Env from the HIV-1_DS-br _isolate, recovered from an adult AIDS patients with dementia by Dr. Suzanne Gartner (Johns Hopkins University) through co-cultivation of brain tissue with monocyte-derived macrophages (MDM) [73,74]. Human adult microglial cells were previously infected with this viral isolate and total DNA was isolated 3 days post-infection and used for *env *amplification [38]. We have now fully sequenced these *env *clones (named DS12, DS13 and DS17) (kindly provided by Dr. Francisco González-Scarano, University of Pennsylvania) (GenBank accession numbers, EU850429–EU850431) and used them for phenotypic analyses to determine whether the Env phenotypes are similar to those identified in *env *amplified directly from brain tissue of HIV-1-infected individuals. In addition, we have used QuikChange site-directed mutagenesis to generate the Env mutant DS17(N283T), in which the N283 residue present in the DS17 clone was mutated to T. The mutation was verified by sequence analysis.

### Peptides and other reagents

Fusion inhibitors T-20 (YTSLIHSLIEESQNQQEKNEQELLELDKWASLWNWF) and the more potent T-1249 (WQEWEQKITALLEQAQIQQEKNEYELQKLDKWASLWEWF) are based on the sequence of the HR2 region of gp41e from the HIV-1_HxB _isolate and inhibit viral entry by targeting the envelope glycoproteins at a fusion intermediate state [75,76]. T-20 and T-1249, as well as the derived peptides T-20-BR (YT**N**LI**YN**LIE**K**SQNQQE**M**NEQELL**K**LD**T**WASLWNWF) and T-1249-BR (WQEWEQKITALLEQAQIQQE**M**NE**Q**ELQKLD**T**WASLWEWF) that contain amino acid changes (shown in bold) present in the HR2 region of the previously characterized H0002GH brain-derived *env *clones [54], were custom synthesized (BioSynthesis, Inc., Lewisville, TX). In addition, a biotinylated, HR1-derived peptide (N40, biotin-RQLLSGIVQQQNNLLRAIEAQQHLLQLTVWGIKQLQARVL) and a biotinylated scramble peptide (N40scr), to be used as negative control in biosensor experiments, were also custom synthesized. All peptides were dissolved to 1–2 mg/ml final concentration in 10–25% DMSO, aliquoted and stored at -80°C until use. The desired dilutions for inhibition or interaction experiments were freshly made in DMEM or phosphate-buffered saline (PBS), respectively.

The gp120-targeted small molecule BMS-378806 [77,78], a gift from Dr. Amos Smith (University of Pennsylvania), is an entry inhibitor that has been proposed to function through inhibition of CD4 binding [79,80]; however, other evidences indicate that it may bind to unliganded gp120 and target the CD4-induced conformational re-arrangement of gp120 and gp41 [81,82]. The 12p1-derived short peptide HNG-105 (kindly provided by Drs. Hosahudya Gopi and Irwin Chaiken, Drexel University College of Medicine) was generated by click conjugation and functions as an entry inhibitor since it prevents viral infection and has been shown to inhibit interactions of both monomeric and trimeric soluble gp120 with soluble CD4 [83-86]. It has been suggested that HNG-105 works through a novel allosteric mechanism, interacting with a site different from the CD4 or co-receptor binding sites but resulting in a lower affinity of gp120 for either of its receptors. The SK3 anti CD4 mAb was purchased from Becton Dickinson (San Jose, CA).

### Pseudotype production and infections

To produce Env-pseudotyped, luciferase-reporter viruses, 293T cells were co-transfected using calcium phosphate precipitation (ProFection Mammalian Transfection System, Promega, Madison, WI) with each Env expression vector and with the Env-deficient, pNL4-3-luc^+^env^- ^provirus, developed by N. Landau by introducing a frameshift mutation in the *env *gene of pNL4-3-luc^+ ^[87]. Culture supernatants containing the pseudotyped particles were collected 48–72 hours after transfection, clarified by centrifugation, aliquoted and stored at -80°C until use. Pseudotype particles lacking envelope glycoproteins, which are generated by co-transfecting the luciferase HIV-1 pro-viral backbone with an empty expression vector, are used as negative control for all pseudotype infections. Pseudotype stocks were quantitated using a p24^gag ^antigen capture ELISA (SAIC-National Cancer Institute) and normalized amounts of stocks were used to infect target cells for 5–6 hours at 37°C. At 2–3 days post-infection, the cells were washed with PBS and lysed, and the amount of entry mediated by each Env was measured by detecting luciferase activity (Luciferase Assay System, Promega) in a microplate luminometer (GloMax, Promega), following manufacturer's instructions. Results are obtained as relative light units (RLU) per second. At least three independent experiments were performed for each analysis described below, with 2–8 replicates within each experiment.

To test for CD4 and CCR5 dependencies, QT6 cells were transfected with 5 or 0.5 μg DNA (per 10^6 ^cells) of the corresponding expression vectors, since it has been shown that these amounts result in either high or low receptor expression levels, respectively, in the cell surface [54]. Empty pcDNA3.1 vector was used to equal the total amount of DNA used per well. After transfection, target cells were collected with Versene (Invitrogen), re-plated into 96-well plates and incubated overnight before infection.

To investigate the avidity of the interaction between Env trimers in the surface of viral particles and CD4 in the target cell membrane, HOS-CD4-CCR5 cells plated in 96-well plates 24 hours before infection, were incubated for 30–60 minutes at 4°C with 2× the final concentration of SK3 anti-CD4 mAb in a volume of 50 μl; subsequently, 50 μl of viral pseudotype stocks were added and the cells were incubated for 5–6 hours at 37°C before removing the inoculum, washing with PBS and incubating for 2–3 days.

To test for sensitivity to fusion inhibitors, BMS-378806 and HNG-105, the medium from HOS-CD4-CCR5 cells plated in 96-well plates 24 hours before infection, was removed and replaced by fresh medium containing 2× the final concentration of the corresponding inhibitor, and immediately the same volume of viral stocks was added. After 5–6 hours at 37°C, inocula and inhibitors were removed and the cells were incubated for 2–3 days before evaluating the amount of infection.

Finally, macrophage tropism was determined by infecting in parallel MDMs and HOS-CD4-CCR5 cells. After 2–3 days, the extent of infection was measured by testing for luciferase activity in cell lysates (Luciferase Assay System, Promega).

### Env-mediated cell-to-cell fusion assays

Cell-to-cell fusion assays were used to test for fusogenicity, receptor utilization and sensitivity to inhibitors. Briefly, 10^6 ^QT6 effector cells per well in 6-well plates were infected at a multiplicity of infection of 2 with the recombinant vaccinia virus vTF1.1 that expresses the bacteriophage T7 RNA polymerase [88] for 1–2 hours at 37°C. After removing the inoculum and washing with PBS, DMEM supplemented with rifampicin was added and the cells were subsequently transfected by calcium phosphate precipitation with an Env expression vector (or empty pcDNA3.1 as a negative control). After 4 hours at 37°C, the cells were washed, DMEM with rifampicin was added, and the cells were incubated overnight at 32°C. Concurrently, 10^6 ^QT6 target cells per well in 6-well plates were transiently transfected with 5 μg DNA of each CD4 and CCR5 expression vectors, or CD4 plus empty vector as negative control, and 3 μg of pT7-luc, a luciferase-expression vector where the firefly luciferase gene is under the control of the T7 promoter, for 5 hours at 37°C. DNA was then removed, and cells were collected with Versene and re-plated into 96-well plates and incubated at 37°C overnight. The next day, effector cells were collected, overlaid onto target cells, and incubated at 37°C for 5–6 hours (for receptor utilization and inhibition experiments), or for various time periods between 30 minutes and 7 hours (for fusogenicity experiments). Luciferase activity (indicative of fusion) was measured in cell lysates as described above. At least two independent experiments were performed for testing the sensitivity to the SK3 anti-CD4 mAb, the T-1249 and T-1249-BR fusion inhibitors and the small molecule BMS-378806, with samples at least in triplicate within each experiment. Results were obtained as RLU per second.

### Optical biosensor binding experiments

Interaction analyses were performed on a Biacore^® ^3000 optical biosensor (GEHC, Biacore, Piscataway, NJ) with simultaneous monitoring of four flow cells. Immobilization of the HR1-based peptides N40 and N40scr (a scramble peptide to be used as negative control that was generated at ) was achieved through interaction of an amino-terminus biotin molecule on the peptides with the streptavidin-coated SA sensor chips (GEHC, Biacore) following standard protocols. The N40scr surface served as a reference.

After immobilization, increasing concentrations of T-1249 and T-1249-BR were passed over the N40 and N40scr surfaces at 25°C using a flow rate of 25 μl min^-1^. Due to the hydrophobic nature of the peptides, 8% DMSO was included in the running buffer (25 mM Tris-HCl, 150 mM NaCl, pH 7.4) to keep them in solution. Regeneration of the peptide surfaces during runs was achieved by pulses of 0.05% SDS, followed by extensive washes of the integrated microfluidics cartridge.

### Statistical analyses

Inhibition curves obtained by plotting the relative infection (expressed as a percent of untreated) versus the concentration of the inhibitor were analyzed by non-linear regression with a sigmoidal dose-response model (variable slopes) using GraphPad Prism software, version 4.02 (GraphPad Software, San Diego, CA); this analysis uses a four parameter logistic equation, Y = Bottom + (Top-Bottom)/($\text{1}+\text{1}0^{\left(({\text{log}}_{\text{1}0}{\text{IC}}_{\text{5}0}-\text{X})\cdot \text{Slope}\right)}$) where Y is the response, Bottom is 0 and Top is 100, X is the logarithm of the concentration, and Slope is the slope of the curve. 50% inhibitory concentrations (IC_50_) and 95% confidence intervals were estimated from the non-linear regression analysis. Fusogenicity data were analyzed by non-linear regression with a one-phase exponential model (using GraphPad Prism) that uses the equation Y=Y_max_(1-e^(-kX)^), where Y_max _is the maximum response and k is the observed rate. CD4 dependence data were analyzed with SPSS, version 16, using the non-parametric Mann-Whitney test.

## Results and discussion

### Construction of chimeric and mutant Env

To investigate the viral determinants for the phenotypic differences reported between the brain- and spleen-derived Env of a patient with HIV-1 associated dementia and encephalitis [54], we constructed chimeric *env *genes as described in Materials and Methods. There were 13 amino acid differences between the wild-type BR and SPL Env clones in V1/V2, 8 in V3 and 9 in V4, in addition to less numerous changes in more conserved Env regions. Six amino acid differences were also found absolutely conserved between all brain and spleen clones from this individual in the HR2 region of gp41e. First, we generated the BS and SB chimeras (brain gp120-spleen gp41 and spleen gp120-brain gp41, respectively) in pcDNA3.1 (Figure 1). These chimeric Env were functional in cell-to-cell fusion assays but, as with the parental wild-type Env, they did not result in a highly efficient production of infectious pseudotypes. To test whether we would be able to produce pseudotype viruses and perform infection experiments utilizing a different expression vector, we sub-cloned most of the gp160 coding regions, except for the first 11 amino acids after the signal peptide in gp120 and the last 132 amino acids in gp41 (which were identical in the brain and spleen derived parental Env), from the BR, SPL, BS and SB *env *clones in pcDNA3.1 into the pHXB2-env vector [72] in which efficient expression is obtained from an SV40 promoter. Therefore, we generated HxB-BR, -SPL, -BS and -SB chimeras, which will be referred to as BR, SPL, BS and SB, respectively. Additional chimeric Env containing the brain V1/V2-C2-V3 fragment in the context of spleen Env and vice versa (Bv1v3 and Sv1v3, respectively) were generated, since numerous amino acid differences between the brain and spleen Env cluster in this gp120 region, which has previously been shown to contain the determinants for increased fusogenicity and low CD4 dependence in the Env of the *in vitro *microglia-adapted isolate HIV-1_Bori-15 _[49,50]. Furthermore, we have also constructed chimeric Env containing only the brain-derived V1/V2 region in the context of the spleen Env and vice versa (Bv1v2 and Sv1v2, respectively), and we have also generated the mutant Env SPL(T283N) and BR(N283T) to evaluate the potential role of N283 vs. T283 in the phenotype of these Env, as described recently by Dunfee et al. [53].

Finally, since there is the potential that the *env *genes amplified by PCR from total DNA isolated from brain tissue might not fully represent a truly replicating virus present in that tissue, we decided to similarly characterize several *env *genes obtained by PCR from microglial cells that had been infected with HIV-1_DS-br _[38], a viral isolate recovered through co-cultivation of brain tissue derived from an adult, demented AIDS patient with normal MDMs [73,74]; in addition, one of these clones (DS17) was mutated to generate DS17(N283T) to test as well the potential role of N283 in an additional Env background.

### Analysis of CD4 and CCR5 dependence

**Figure 2:**
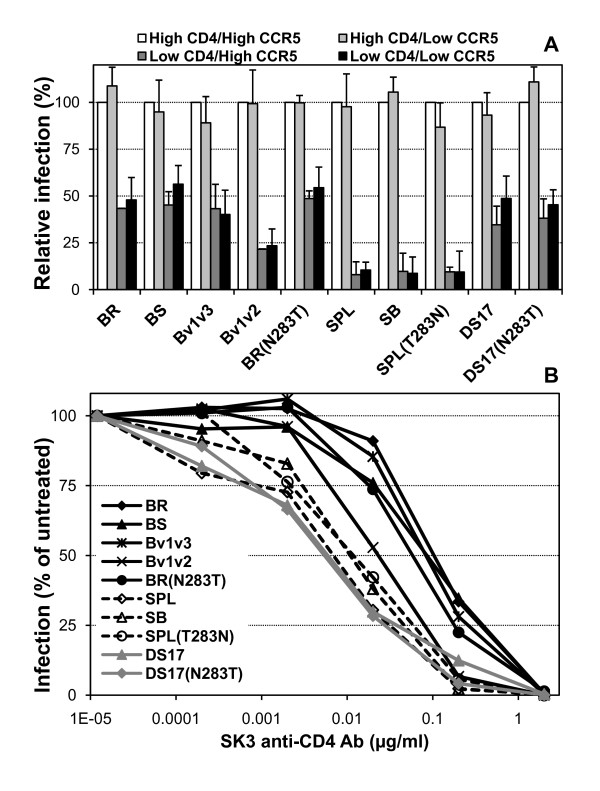
**CD4 dependence and avidity of pseudotypes containing wild-type, chimeric and mutant Env**. Env-pseudotyped viruses were used to infect transiently-transfected QT6 cells expressing either low or high levels of CD4 and CCR5 (top) or HOS-CD4-CCR5 cells in the presence of the SK3 anti-CD4 mAb (bottom). BR, BS, Bv1v3 and BR(N283T) showed greater ability to infect cells expressing low levels of CD4 (low CD4 dependence) and were less sensitive to inhibition by the anti-CD4 mAb SK3 than SPL, SB and SPL(T283N), while Bv1v2 had an intermediate phenotype. The brain isolate-derived DS17 Env and the DS17(N283T) mutant had a mixed phenotype with low CD4 dependence but high sensitivity to the anti-CD4 mAb. Average + standard error from 4 independent experiments (top) and data from a representative experiment repeated at least 4 times with similar results (bottom) are shown.

First, we produced pseudotypes as indicated in Materials and Methods and evaluated CD4 and CCR5 dependence using target cells transiently transfected with various amounts of the corresponding expression plasmids, which results in the expression of low or high levels of CD4 and CCR5, as described previously [54]. In addition, as a surrogate for gp120:CD4 affinity, we evaluated the inhibition of infection by the SK3 anti-CD4 mAb in HOS-CD4-CCR5 cells that stably express very high levels of CD4 and CCR5. As expected, the BS and SB chimeras – containing brain gp120-spleen gp41 and spleen gp120-brain gp41, respectively – each displayed the same phenotype of the corresponding wild-type BR and SPL Env, since CD4 dependence is mostly determined by the gp120 subunit. Thus, BR and BS showed greater ability to use low levels of CD4 for infection (or lower CD4 dependence) (Figure 2A) and reduced sensitivity to inhibition of infection by the anti-CD4 mAb (Figure 2B) than SPL and SB. In addition, the Bv1v3 chimera and the BR(N283T) mutant displayed a similar phenotype to BR and BS, with low CD4 dependence and reduced sensitivity to the anti-CD4 mAb, while the phenotype of the SPL(T283N) mutant was identical to that of SPL and SB. Finally, the Bv1v2 chimeric Env showed an intermediate ability to use low levels of CD4 for infection and a sensitivity to inhibition by the anti-CD4 mAb that was closer to the wild-type parental SPL than to the BR Env (Figure 2).

As shown in Table 1, statistical analysis of relative infection in the presence of low levels of CD4, irrespective of the levels of CCR5, using the non-parametric Mann-Whitney test (SPSS), indicated that BR, BS, Bv1v3 and BR(N283T) all had statistically significant higher infectivity than SPL, SB and SPL(T283N), as well as than Bv1v2 (except for Bv1v3).

**Table 1:** Analysis of the relative pseudotype infection in cells expressing low levels of CD4.

	BS	Bv1v3	Bv1v2	Br (N283T)	Spl	SB	Spl (T283N)	DS17	DS17 (N283T)
Br	0.670	0.462	**0.019**	0.831	**0.019**	**0.020**	**0.021**	0.394	0.394

BS		0.715	**0.025**	0.522	**0.010**	**0.010**	**0.011**	0.262	0.337

Bv1v3			0.144	0.584	**0.018**	**0.049**	**0.027**	0.465	0.715

Bv1v2				**0.016**	0.109	0.054	0.055	0.631	0.337

Br(N283T)					**0.004**	**0.010**	**0.011**	0.200	0.423

Spl						0.669	0.670	**0.037**	**0.037**

SB							0.767	0.087	0.054

Spl(T283N)								0.055	0.055

DS17									0.873

Significance (*p*) values were obtained with the non-parametric Mann-Whitney test for the comparison of the relative infection with the indicated pseudotypes in cells expressing low levels of CD4; *p *values below 0.05 were considered significant (shown in bold).

For the anti-CD4 mAb inhibition data, non-linear regression analysis showed that all curves fitted well to the sigmoidal dose-response model (R^2 ^≥ 0.92) and that the null hypothesis of a single best fit curve for all data sets could be rejected (F test, p < 0.0001). We then compared all possible pairs and found that dose-response curves for BR, BS, Bv1v3 and BR(N283T) were statistically different from the curves for SPL, SB, SPL(T283N) and Bv1v2, since the null hypothesis of a single best fit curve for the two data sets could be rejected (Table 2). The analysis provided as well with estimated 50% inhibitory concentrations (IC_50_) and 95% confidence intervals that are shown in Table 3.

**Table 2:** Analysis of inhibition of pseudotype infection in the presence of anti-CD4 antibody.

	BS	Bv1v3	Bv1v2	Br (N283T)	Spl	SB	Spl (T283N)	DS17	DS17 (N283T)
Br	0.2363	0.8349	**0.0032**	0.3344	**0.0005**	**0.0004**	**0.0027**	**<0.0001**	**<0.0001**

BS		0.2286	**0.0010**	0.2638	**<0.0001**	**<0.0001**	**<0.0001**	**<0.0001**	**<0.0001**

Bv1v3			**0.0021**	0.5091	**<0.0001**	**0.0001**	**0.0010**	**<0.0001**	**<0.0001**

Bv1v2				**0.0472**	**0.0212**	0.1048	0.1863	**0.0002**	**0.0001**

Br(N283T)					**0.0008**	**0.0013**	**0.0064**	**<0.0001**	**<0.0001**

Spl						0.1783	0.1787	0.7203	0.8227

SB							0.8959	**0.0055**	**0.0066**

Spl(T283N)								**0.0067**	**0.0023**

DS17									0.1487

Significance (*p*) values were obtained with an F test; *p *values below 0.05 were considered significant (shown in bold) an indicate rejection of the null hypothesis of a single best fit curve for both data sets.

**Table 3:** IC_50 _values obtained in inhibition experiments^a^.

	SK3 anti-CD4 (ng/ml)	T-1249 (nM)	T-1249-BR (nM)	BMS-378806 (nM)	HNG-105 (μM)
Br	119 (58–246)	173 (129–230)	195 (157–298)	15 (9.4–23)	3.8 (2.2–6.5)

BS	79 (60–102)	140 (113–172)	155 (83–289)	20 (13–31)	1.8 (0.9–3.7)

Bv1v3	94 (55–158)	227 (110–468)	245 (156–387)	8.2 (5.7–12)	21 (12–36)

Bv1v2	22 (13–36)	224 (174–288)	155 (123–195)	4.8 (3.5–6.6)	48 (9.8–231)

Br(N283T)	60 (32–114)	166 (102–271)	221 (148–329)	13 (4.6–35)	7.2 (2.2–24)

Spl	5.7 (2.3–14)	37 (19–71)	27 (17–43)	1.8 (1.3–2.7)	16 (6.7–39)

SB	11 (6.4–18)	83 (53–129)^b^	41 (30–56)^b^	2.0 (1.2–3.5)	25 (12–50)

Sv1v3	n.d.	n.d.	n.d.	0.9 (0.6–1.3)	n.d.

Sv1v2	n.d.	n.d.	n.d.	1.2 (0.7–2.2)	n.d.

Spl(T283N)	11 (7.3–18)	n.d.	n.d.	0.9 (0.7–1.3)	n.d.

DS17	5.4 (3.8–7.7)	210 (160–274)^b^	92 (73–116)^b^	41 (29–58)	4.1 (2.7–6.2)

DS17(N283T)	5.1 (4.0–6.4)	155 (119–202)	104 (72–150)	32 (24–41)	2.6 (1.0–6.6)

BaL	n.d.	102 (85–122)	124 (103–149)	29 (15–59)	58 (14–241)

HxB	n.d.	n.d.	n.d.	3.9 (2.8–5.4)	1.6 (0.7–3.6)

^a ^Non-linear regression analysis of dose response curves generated for various viruses and inhibitors was performed with GraphPad Prism software; IC_50 _values are shown, with 95% confidence limits between parenthesis.

^b ^SB and DS17 were the only Env that demonstrated statistically significant greater sensitivity to T-1249-BR than to parental T-1249 (F test, p < 0.05 and p < 0.001, respectively).

n.d., not determined.

Surprisingly, the *env *clones obtained from genomic DNA extracted from microglial cells infected with an isolate recovered from brain tissue co-cultured with MDMs (DS12, DS13 and DS17) [38,73,74] displayed a mixed phenotype, since they all showed a similar ability than BR to infect cells expressing low levels of CD4 but had a much greater sensitivity, similar to that observed with SPL, to inhibition of infection by the SK3 anti-CD4 mAb (Figure 2 and Tables 2 and 3; only DS17 is shown but the other two clones had the same phenotype). In addition, the mutant Env generated in the background of the DS17 *env *clone, DS17(N283T), had an identical phenotype to the parental DS17 Env in both CD4 dependence and sensitivity to anti-CD4 mAb. Thus, while some Env with a greater capacity to utilize low levels of CD4 for infection may also feature a high avidity for CD4 (as shown by the ability to outcompete the presence of an anti-CD4 mAb), some other Env may be able to acquire low CD4 dependence by a different mechanism not involving an increased avidity for CD4 in the context of the trimeric Env:CD4 interaction.

Although it has been reported that brain-derived Env may also have reduced CCR5 dependence and/or increased affinity for CCR5, albeit in the absence of differences in sensitivity to the CCR5 inhibitor TAK-779 [55], we did not find such phenotypic differences in our initial characterization of these brain- and spleen-derived Env [54]. Concurrently, we did not observe any difference in CCR5 dependence among the wild-type, chimeric and mutant Env evaluated in this study. In addition, contrary to CD4, CCR5 levels in microglia and macrophages seem to be comparable to those in other target cells for HIV-1 such as CD4^+ ^T-cells [30,33,89,90], and therefore it may not be surprising that acquisition of reduced CCR5 dependence by neurotropic Env may occur less frequently than that of low CD4 dependence.

### Fusogenicity

In addition to using single-round, Env-pseudotyped viruses to test for the ability of various Env to mediate infection, Env-mediated cell-to-cell fusion assays are also commonly used to evaluate their ability to interact with receptors and mediate fusion in the context of cell-cell interaction, rather than virus-cell interaction. We evaluated CD4 dependence and sensitivity to the SK3 anti-CD4 mAb with wild-type, chimeric and mutant Env using cell-to-cell fusion and obtained similar results to those described above with pseudotype infection (data not shown), confirming similar functionality in both types of assays. The presence of multinucleated giant cells is the principal neuropathological finding of HIV-1 infection in the brain and the hallmark of HIV-1 encephalitis [24-27], and high fusion was found to be characteristic of the Env of an *in vitro *microglia-adapted isolate [50,51] and of *env *clones derived from brain tissues [54,55,57]. In addition, asparagine in position 362 in gp120's C3 region, which has been found in a higher proportion of CCR5-using Env derived from AIDS patients than from individuals with asymptomatic infection, has been shown to contribute, albeit in a strain-dependent manner, to increased fusogenicity and higher sensitivity to an anti-CD4bs mAb, perhaps by promoting greater exposure of the CD4bs and/or stabilizing the CD4-bound Env conformation [91]. However, wild-type BR and SPL Env, as well as DS12, DS13 and DS17, all have N362 and thus any difference in fusogenicity will be due to other determinants.

**Figure 3:**
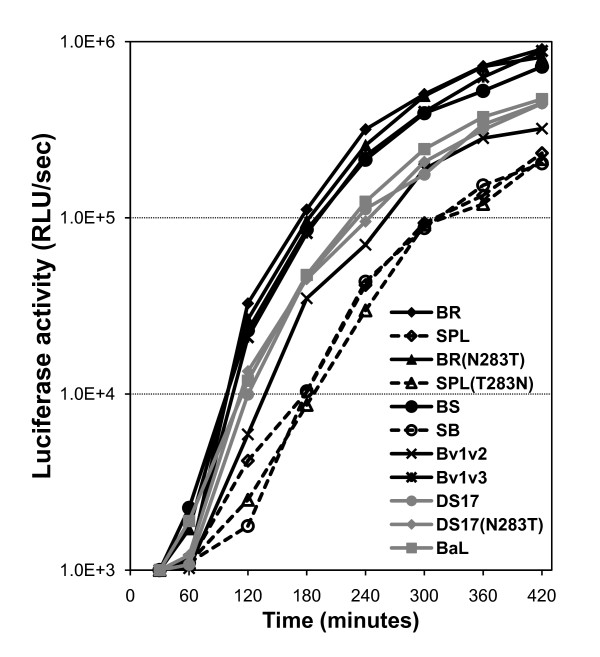
**Fusogenicity of wild-type, chimeric and mutant Env**. Cell-to-cell fusion assays of Env-expressing effector QT6 cells and target QT6 cells transiently transfected for the expression of high levels of CD4 and CCR5 were performed to test fusogenicity. Co-culture of effector and target cells for 30–420 minutes showed that BR, BR(N283T), BS and Bv1v3 fused more efficiently than SPL, SPL(T283N) and SB, while Bv1v2, DS17, DS17(N283T) and BaL showed an intermediate phenotype. Data shown is the average background-subtracted, actual relative light units (RLU) per second from 3 independent experiments.

Therefore, we decided to test the fusogenicity of wild-type, chimeric and mutant Env in a cell-to-cell fusion assay in which the co-cultivation of effector and target cells was performed for 30–420 minutes, to obtain information regarding the amount of fusion mediated by each Env at every time point. Background-subtracted luciferase activity (used as reporter gene) data presented in Figure 3 demonstrated that BR, BR(N283T), BS and Bv1v3 mediated fusion more efficiently than SPL, SPL(T283N) and SB, while Bv1v2, DS17, DS17(N283T) and BaL (a well-characterized, macrophage-tropic Env [92] used as a control) showed an intermediate level of fusion.

To analyze these results, we only considered the fusogenicity data between 60–420 minutes, since this assay is based on a reporter gene and there is of course a delay between the completion of fusion and the production of luciferase. However, this delay should be the same for all Env, and differences observed in luciferase activity should therefore reflect differences in fusogenicity, that is, in the amount and rate of fusion mediated by each individual Env. All curves fitted well to the exponential model in non-linear regression analysis (R^2 ^≥ 0.96) and the null hypothesis of a single best curve for all data sets could be rejected (F test, p < 0.0001). As shown in Table 4, we estimated the amount of time needed for each Env to cause the luciferase activity in cell lysates to reach two arbitrary thresholds, 1.0E+4 and 1.0E+5 RLU/second, and found that, in agreement with the differences in fusion efficiency indicated above, BR, BR(N283T), BS and Bv1v3 were able to reach these thresholds in a remarkably shorter time than SPL, SPL(T283N) and SB, while again Bv1v2, DS17, DS17(N283T) and BaL showed an intermediate phenotype. Similarly, the time needed for the luciferase activity to increase from the lower to the higher threshold reflected the higher fusogenic capacity of BR, BR(N283T), BS and Bv1v3 when compared to SPL, SPL(T283N) and SB, since the former required approximately half the time than the later (Table 4). Therefore, in this group of wild-type, chimeric and mutant Env high fusogenicity mostly correlates with low CD4 dependence and high avidity for CD4. However, DS17 and DS17(N283T) displayed a mixed phenotype since they had intermediate fusogenicity with low CD4 dependence but a lack of increased avidity for CD4 (as indicated by the sensitivity to inhibition by the anti-CD4 mAb).

**Table 4:** Analysis of Env-mediated fusogenicity^a^.

	Log_10 _[Y_max_] (95% confidence limit)	Time to reach 1.0E+4 RLU/sec^b^	Time to reach 1.0E+5 RLU/sec^b^	Difference
Br	5.94 (5.76–6.12)	99	163	64

Spl	5.43 (5.01–5.85)	161	307	146

BS	5.83 (5.65–6.01)	106	178	72

SB	5.59 (5.49–5.70)	175	312	137

Br(N283T)	5.93 (5.74–6.12)	104	171	67

Spl(T283N)	5.50 (5.12–5.88)	175	323	148

Bv1v3	5.93 (5.71–6.14)	109	180	71

Bv1v2	5.62 (5.43–5.81)	137	243	106

DS17	5.65 (5.44–5.86)	125	220	95

DS17(N283T)	5.61 (5.32–5.89)	121	215	94

BaL	5.70 (5.51–5.89)	122	211	89

^a^Non-linear regression analysis was performed using an exponential model (Y_max_, estimated maximal response in RLU/second) with background-subtracted data comprising 60–420 minutes after initiation of co-culture.

^b^Estimated time is expressed in minutes.

### Sensitivity to fusion inhibitors

**Figure 4:**
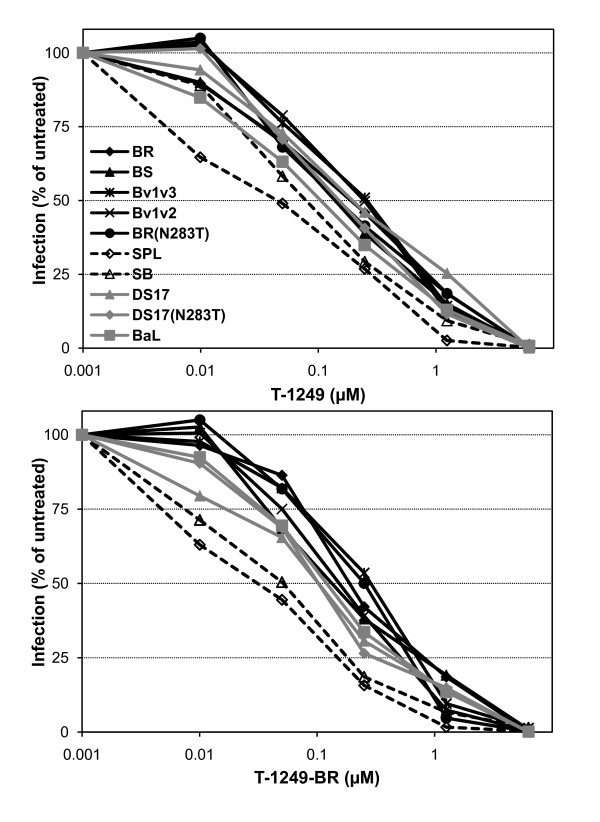
**Sensitivity of pseudotypes to fusion inhibitors**. Pseudotypes were incubated for 1 h. at 37°C with fusion inhibitors T-1249 (A) and T-1249-BR (a peptide that contains amino acid residues conserved in brain but absent in spleen derived Env of one individual) (B), and then the mixture was used to infect HOS-CD4-CCR5 cells. Pseudotypes containing BR, BS, Bv1v3, Bv1v2 and BR(N283T) Env were significantly less sensitive to T-1249 and T-1249-BR than those with SPL and SB Env. Data from a representative experiment repeated at least 4 times with similar results are shown.

We had previously described that the increase in fusogenicity of brain-derived Env from this individual correlated with reduced sensitivity to the fusion inhibitor T-1249 [54]. These clones from brain contained several conserved amino acid differences in the HR2 region of gp41 with respect to those from the spleen, at positions with various degrees of polymorphism. Although the high fusogenicity of the BS and Bv1v3 but not the SB chimeras seems to indicate that these changes in gp41 do not determine the fusogenicity observed, it would still be possible that they play a role in the reduced sensitivity to fusion inhibitors, e.g., by promoting an improved interaction between HR1 and HR2 that might lead to a more efficient fusion process and/or reduced sensitivity to inhibitors. For this reason, we wanted to test whether fusion inhibitors-derived peptides containing all or some of these conserved changes found in brain-derived gp41 could function as inhibitors of the fusion process with similar or greater potency than T-20 and T-1249. Thus, T-20 (also known as Enfuvirtide) and T-1249, as well as derived peptides T-20-BR (YT**N**LI**YN**LIE**K**SQNQQE**M**NEQELL**K**LD**T**WASLWNWF) and T-1249-BR (WQEWEQKITALLEQAQIQQE**M**NE**Q**ELQKLD**T**WASLWEWF) that contain amino acid changes present in the HR2 region of H0002GH brain-derived Env (shown in bold), were synthesized and used for inhibition of infection by pseudotypes containing wild-type, chimeric and mutant Env. All pseudotypes showed very similar inhibition by the less potent T-20 and derived peptide T-20-BR (data not shown). However, inhibition experiments with T-1249 and T-1249-BR (Figure 4) demonstrated that pseudotypes with BR, BS, Bv1v3, Bv1v2 and BR(N283T) Env, as well as those with DS17, DS17(N283T) and BaL, were less sensitive than those with SPL and SB Env. Non-linear regression analysis and estimation of IC_50_s confirmed the differences in sensitivity (Table 3). Therefore, these data suggested that the gp41 subunit of the brain-derived Env is not responsible for the reduced sensitivity to fusion inhibitors. On the contrary, it seems that the gp120 subunit of the brain Env, and specifically the regions involved in conferring low CD4 dependence, are also involved in determining the increased fusogenicity and reduced sensitivity to fusion inhibitors, at least in these Env genetic backgrounds.

In addition, most pseudotypes showed similar sensitivity to both T-1249 and T-1249-BR, suggesting that the fusion inhibitor modified with amino acids from brain Env's HR2 region does not usually have higher efficiency than parental T-1249. However, as shown in Table 3, the SB and DS17 pseudotypes had statistically significant greater sensitivity to T-1249-BR than to T-1249, since an F test performed as part of non-linear regression analysis rejected the null hypothesis of a single best fit curve for both inhibitors with each pseudotype (p < 0.05 and p < 0.001, for SB and DS17, respectively). Thus, it is interesting that only in the context of the SB chimera (but not with the wild-type brain-derived Env) and DS17, T-1249-BR seemed to show a slightly, but significant, higher potency than parental T-1249. Since the SB chimera does not have low CD4 dependence, high avidity for CD4 or increased fusogenicity, but does contain the brain gp41 subunit in which the T-1249-BR sequence is based upon, it is possible that only in this context of limited efficiency in the interaction with CD4 and limited fusogenicity, T-1249-BR will be able to demonstrate a greater efficiency than T-1249 at blocking fusion mediated by the brain-derived gp41.

### Interaction between HR1- and HR2-derived peptides

In addition, we developed a surface plasmon resonance assay to evaluate whether the modified T-1249-BR might differ from parental T-1249 in the interaction with the HR1 region in gp41. In this assay, a biotinylated, HR1-derived peptide (N40) was synthesized and immobilized onto the surface of a streptavidin-coated chip to evaluate binding of the HR2-derived T-1249 and T-1249-BR using a Biacore^® ^3000 optical biosensor. Under the experimental conditions outlined in Materials and Methods, and over a concentration range of 81–650 nM, both HR2-derived peptides interacted in a similar manner with the immobilized HR1-derived peptide resulting in an almost identical amount of mass bound at the sensor surface (1 response unit = 1 pg/mm^2^). Moreover, the interaction was specific since no binding was observed to the immobilized, biotinylated N40scr, scramble peptide (data not shown). Thus, we did not find a qualitatively appreciable difference between the two peptides.

In summary, the amino acid changes found in the HR2 region of gp41 in brain-derived Env from H0002GH did not seem to affect the interaction between HR2- and HR1-derived peptides in biosensor experiments, and did not seem to contribute to the increased fusogenicity or to the reduced sensitivity to fusion inhibitors, as compared to the spleen-derived Env. Furthermore, low CD4 dependence and reduced sensitivity to the anti-CD4 mAb, and increased fusogenicity and reduced sensitivity to peptide fusion inhibitors seem to be determined by the same viral determinants in gp120.

### Sensitivity to the entry inhibitors BMS-378806 and HNG-105

**Figure 5:**
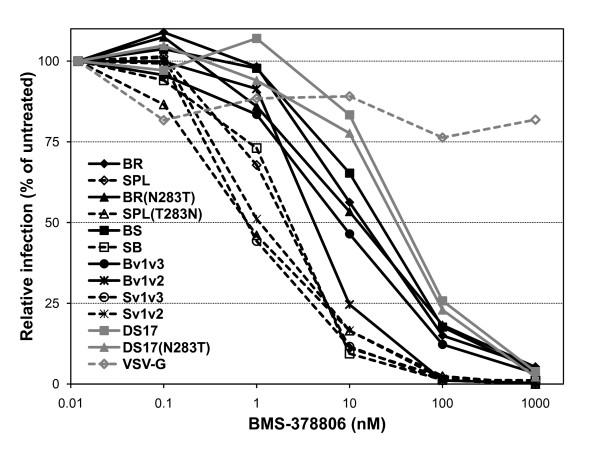
**Sensitivity of pseudotypes to the entry inhibitor BMS-378806**. Pseudotypes were incubated for 1 h. at 37°C with the gp120-targeted small molecule BMS-378806 and then the mixture was added to HOS-CD4-CCR5 cells. Pseudotypes containing BR, BR(N283T), BS and Bv1v3 Env were significantly less sensitive than those with SPL, SPL(T283N), SB, Sv1v3 or Sv1v2, while Bv1v2 displayed an intermediate sensitivity. DS17 and DS17(N283T) did not differ between them but showed reduced sensitivity to BMS-378806 as compared to the BR and Env with related phenotype. Data from a representative experiment repeated at least 4 times with similar results are shown.

To gain additional information regarding the phenotypes of Env with low CD4 dependence and increased fusogenicity, we tested the sensitivity of pseudotype infection to the gp120-targeted entry inhibitors BMS-378806 and HNG-105. The mechanism of action of the small molecule BMS-378806 [77,78] remains controversial; it has been suggested that it suppresses infection by inhibition of CD4 binding [79,80] and/or affecting the CD4-induced conformational re-arrangement of gp120 and gp41 [81,82]. As shown in Figure 5 and Table 5, pseudotypes containing the BR, BR(N283T), BS and Bv1v3 Env were significantly less sensitive to inhibition by BMS-378806 than those with SPL, SPL(T283N), SB, Sv1v3 or Sv1v2 (as demonstrated by non-linear regression analysis with a sigmoidal dose-response model and estimation of IC_50 _values), while Bv1v2 displayed again an intermediate sensitivity. Estimated IC_50 _values are shown in Table 3. This pattern of sensitivity seems to indicate that the low CD4 dependence either modulates or correlates with the sensitivity of various Env to this entry inhibitor. One potential mechanism could be by accelerating the entry process and reducing the time when the Env remains sensitive to inhibition. Alternatively, the low CD4 dependence may be acquired through an altered gp120 conformation that might mainly affect the positioning of the variable loops V1/V2 and V3, perhaps resulting in diminished interaction with BMS-378806. In fact, the V1/V2 variable loop and N-linked carbohydrates on the V1/V2 stem have been shown to influence sensitivity to this small molecule [82]. This latter possibility would also be in agreement with the altered gp120 conformation that was proposed as the mechanism of acquisition of low CD4 dependence for the Env of the previously characterized *in vitro *microglia-adapted isolate, HIV-1_Bori-15 _[49,50,52].

**Table 5:** Analysis of inhibition of pseudotype infection by BMS-378806

	Spl	BS	SB	Bv1v3	Bv1v2	Sv1v3	Sv1v2	Br (N283T)	Spl (T283N)	DS17	DS17 (N283T)	BaL	HxB
Br	**<0.001**	0.598	**<0.001**	**0.044**	**<0.001**	**<0.001**	**0.002**	0.620	**<0.001**	**0.003**	0.107	0.456	**0.002**

Spl		**<0.001**	0.916	**<0.001**	**0.001**	0.239	0.521	**<0.001**	0.162	**<0.001**	**<0.001**	**<0.001**	**0.010**

BS			**<0.001**	**0.003**	**<0.001**	**<0.001**	**<0.001**	0.392	**<0.001**	**0.040**	0.422	0.688	**<0.001**

SB				**<0.001**	**0.039**	0.390	0.610	**0.002**	0.323	**<0.001**	**<0.001**	**0.003**	0.090

Bv1v3					**<0.001**	**0.001**	**0.002**	0.608	**<0.001**	**<0.001**	**<0.001**	**0.047**	**0.044**

Bv1v2						**<0.001**	**0.002**	**0.014**	**<0.001**	**<0.001**	**<0.001**	**<0.001**	**0.006**

Sv1v3							0.456	**0.044**	0.115	**<0.001**	**<0.001**	**<0.001**	**<0.001**

Sv1v2								0.093	0.196	**<0.001**	**<0.001**	**<0.001**	**0.003**

Br(N283T)									0.059	**0.016**	0.193	0.722	0.141

Spl(T283N)										**<0.001**	**<0.001**	**<0.001**	**<0.001**

DS17											0.457	0.368	**<0.001**

DS17(N283T)												0.338	**<0.001**

BaL													**<0.001**

Significance (*p*) values were obtained with an F test; *p *values below 0.05 were considered significant (shown in bold) an indicate rejection of the null hypothesis of a single best fit curve for both data sets.

**Figure 6:**
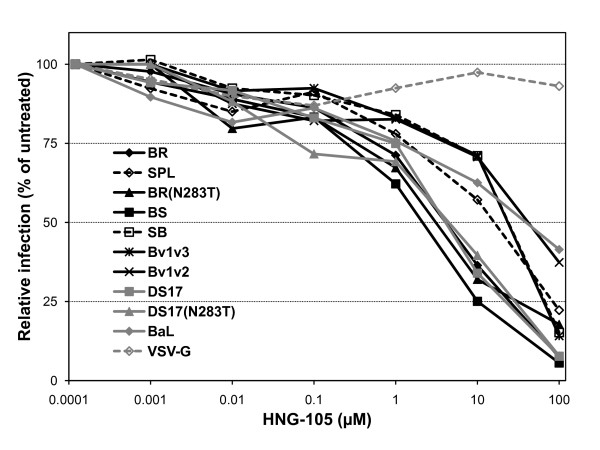
**Sensitivity of pseudotypes to the entry inhibitor HNG-105**. Pseudotypes were incubated for 1 h. at 37°C with the gp120-targeted short peptide HNG-105 and then the mixture was added to HOS-CD4-CCR5 cells. Pseudotypes containing BR, BR(N283T) and BS Env were significantly more sensitive than those with SPL, SB, Bv1v3 or Bv1v2. DS17 and DS17(N283T) did not differ between them and showed similar sensitivity to HNG-105 than BR, BR(N283T) and BS. Data from a representative experiment repeated at least 3 times with similar results are shown.

HNG-105 is an entry inhibitor that, similar to its parental peptide 12p1 [85,86], seems to work by a novel allosteric mechanism that involves interaction with a site in gp120 different than the CD4 or co-receptor binding sites, but results in reduced affinity of gp120 for both receptors [84]. We recently reported that a broad range of sensitivities to HNG-105 could be found not only among Env from various subtypes, but also within subtype B [83], and even between the Env of the highly related parental HIV-1_Bori _and *in vitro *microglia-adapted HIV-1_Bori-15_, which only differ in 8 amino acids [50]. However, these differences in sensitivity to inhibition of infection in the context of pseudotyped viruses did not seem to correlate with changes in direct binding affinity between the soluble monomeric gp120s and HNG-105 in biosensor experiments [83]. Therefore, it is possible that conformational masking of the HNG-105 binding site, which is thought to reside within the gp120 inner domain, in the context of the viral trimers present in the surface of virions could play a principal role in modulating the sensitivity to HNG-105. We thus evaluated wild-type, chimeric and mutant Env and found that BR, BS, BR(N283T), DS17 and DS17(N283T) had greater sensitivity to inhibition than SPL, SB, Bv1v3, Bv1v2 and BaL (Figure 6). Surprisingly, the sensitivity to HNG-105 of Bv1v3 and Bv1v2 is more similar to that of SPL rather than BR, suggesting that the conformational alterations may have differential effects on the accessibility of various inhibitors to their binding sites, and that other regions in the spleen Env may determine the reduced sensitivity to HNG-105. A potential binding site for 12p1 and HNG-105 within gp120 has been identified using mutated Env in pseudotype infection assays; when mutated to alanine, residues K97, E102 and R476 seemed to confer a reduced sensitivity to inhibition by these peptides [85], and it was hypothesized that this may occur by decreasing the affinity of the peptides for the Env complex. However, whether these residues actually represent or belong to a bona fide binding site or not, remains to be elucidated. In addition, all of these foot-print residues are present in both wild-type BR and SPL Env. Thus, we hypothesize that the differences in sensitivity to inhibition of entry by HNG-105 may be caused by conformational variations between the Env, rather than diminished interaction with the inhibitor. These conformational variations in the context of the Env trimers could restrict the accessibility of HNG-105 to its binding site, probably resulting in the altered sensitivity to its inhibitory effect.

In this regard, it has been shown in a very recent paper by Liu et al [93] that changes associated with CD4-binding seem to open up the trimeric Env structure significantly, and this occurs primarily by conformational changes affecting the variable domains. In the context of the sensitivity to HNG-105, a slightly more "triggered" conformation in the BR Env might allow access of the peptide to the inner domain, whereas with the SPL Env, the more "closed" and compact structure would not allow it, perhaps resulting in the differences in sensitivity that we have observed. Considering the dynamism and flexibility of gp120, this could be explained by the possibility that a gp120 that is further away from the CD4-bound conformation would sample CD4-bound conformations less frequently than another gp120 that is closer or partially triggered.

### Macrophage tropism

**Figure 7:**
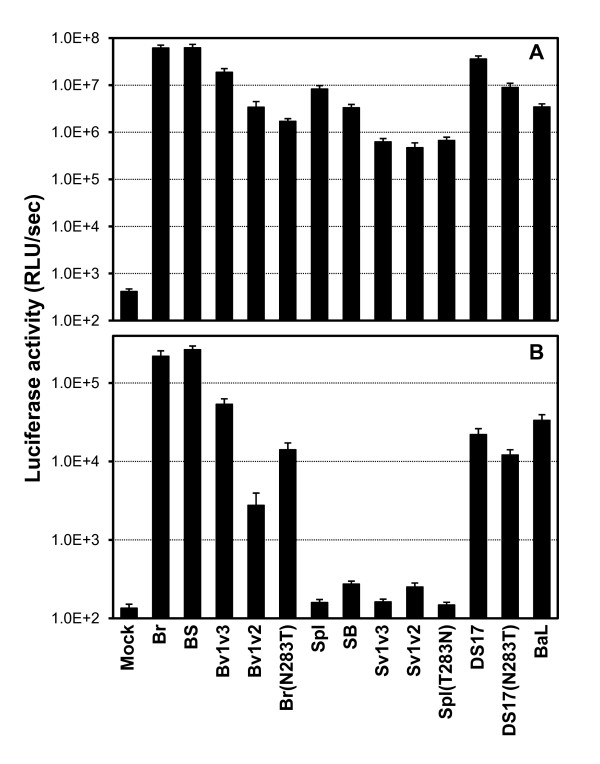
**Pseudotype infection of HOS-CD4-CCR5 cells (top) and monocyte-derived macrophages (bottom)**. Pseudotypes containing wild-type, chimeric and mutant Env that had shown low CD4 dependence and high avidity for CD4, as well as reduced sensitivity to fusion inhibitors and BMS-378806, showed statistically significant greater macrophage tropism than those without low CD4 dependence and less avidity for CD4. Results shown are the mean relative light units (RLU) per second ± standard error of 3 independent experiments (each performed at least in triplicate).

In order to evaluate whether a relationship between the degree of low CD4 dependence and/or increased fusogenicity of wild-type, chimeric and mutant Env, and their ability to mediate infection of primary macrophages (or macrophage tropism) could be established, MDMs were infected with equivalent amounts of pseudotype stocks. HOS-CD4-CCR5 cells were infected in parallel. After 2–3 days, the extent of infection was measured by luciferase activity in cell lysates. As shown in Figure 7, HOS-CD4-CCR5 cells were infected to a similar extent by all pseudotypes, with luciferase activity at least 1000-fold above background levels (mock infection refers to supernatants containing viral particles lacking Env produced by cells co-transfected with the Env-deficient luciferase backbone and an empty vector). By contrast, only those pseudotypes with low CD4-dependent Env showed the ability to infect MDMs, although with various efficiencies. Infection with wild-type BR and BS chimera resulted in luciferase activities 1000-fold above mock infection, and greater than a 100-fold increase was observed with the Bv1v3 chimera, the BR(N283T) mutant and the DS17 and DS17(N283T). The Bv1v2 chimera containing the V1/V2 region of the brain Env in the context of the spleen Env mediated infection of macrophages to a lower extent than BR or Bv1v3, although luciferase levels were still 20-fold above background, indicating that brain Env's V3 region plays a role, together with the V1/V2, in increasing macrophage tropism. However, the Sv1v2 chimera containing the V1/V2 region from SPL and the remaining of the Env from BR (including the V3 loop) failed to mediate any infection of macrophages suggesting that, by itself, the brain Env's V3 region does not confer macrophage tropism.

Therefore, low CD4 dependence seems to correlate not only with fusogenicity and with the sensitivity to fusion and other entry inhibitors, but also with the macrophage tropism of Env. This is in agreement with recent work by Thomas et al. [57] reporting that Env conferring macrophage tropism display low CD4 dependence and higher efficiency of fusion. Our results are also comparable to those recently published by Peters et al. [56] in that there is a relationship between macrophage tropism and sensitivity to certain entry inhibitors. Similar to us, they found that decreasing sensitivity to an anti-CD4 mAb and to BMS-378806 correlated with increasing macrophage tropism. However, they did not find a reduced sensitivity of macrophage-tropic Env to the fusion inhibitor T-20 (in fact, a trend towards increased sensitivity to T-20 with increasing macrophage tropism was observed), while in our study, we did find that macrophage-tropic Env had significantly lower sensitivity to the more potent, second-generation fusion inhibitor T-1249 (but not to T-20), than non-macrophage-tropic Env. It is possible that this discrepancy could be due to the difference in potency between the two fusion inhibitors.

### Modeling studies

**Figure 8:**
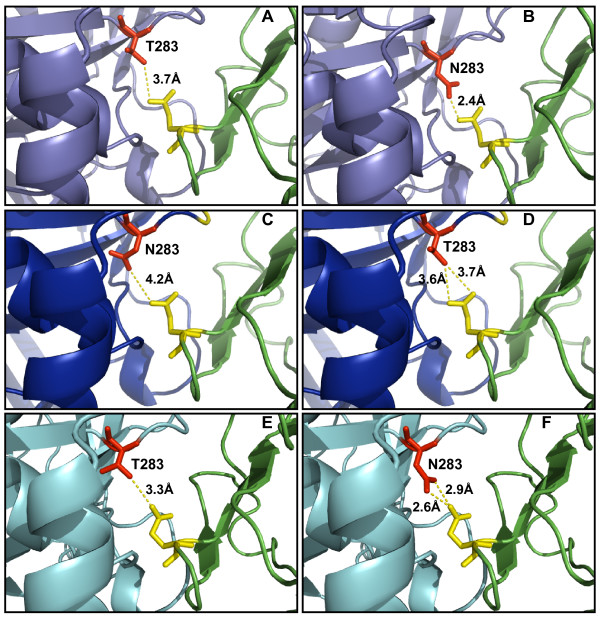
**Modeling the potential role of N283/T283 in various Env for gp120:CD4 interaction**. Swiss PDB Viewer was used to change T283 (A) to N283 (B) in the HIV-1_JR-FL _gp120 crystal structure (2B4C) [95], as previously described [53]; potential hydrogen bonds are indicated by dotted lines and the estimated distance is shown, suggesting a greater likelihood for formation of a hydrogen bond with Q40 (shown in yellow) in CD4 (green) for N283 than for T283, in the HIV-1_JR-FL _gp120 background. DS17 and DS17(N283T) Env were modeled based on the HIV-1_JR-FL _gp120 crystal structure (C and D, respectively) using the Swiss Model Server, and the models showed that the distance between T283 and Q40 would be shorter than between N283 and Q40, although both would be larger than what it is usually accepted for the formation of a hydrogen bond (3.3Å). Finally, the SPL and SPL(T283N) Env were best modeled based on the HIV-1_YU-2 _gp120 crystal structure (E and F, respectively), and the models showed that both could potential make hydrogen bond contacts with Q40 in CD4, although the shorter distance in SPL(T283N) would suggest a greater likelihood for this to occur for mutated N283 than for wild-type T283. The brain-derived Env did not result in a good model with any of the published gp120 crystal structures.

Contrary to Dunfee et al. [53], we did not observe a role for N283 in determining macrophage tropism in these particular Env backgrounds, since the T283N change did not confer macrophage tropism to the SPL Env and the N283T change did not alter the macrophage tropism of the BR or DS17 Env. Therefore, since these changes did not affect macrophage tropism or CD4 dependence (as described above), we performed modeling studies using the Swiss Model Server [94] to gain an insight into the potential differential effects of N or T at position 283 in gp120's C2 region among the various Env utilized in this study. The consensus amino acid of subtype B Env at position 283 is T, and this is the residue found in the HIV-1_JR-FL _gp120, whose structure in complex to CD4 and the CCR5 surrogate 17b mAb is shown in Figure 8A (2B4C; visualized with Pymol v0.99 [DeLano Scientific]; CD4 is shown in green) [95]. As reported before in Dunfee et al. [53], substitution of T for N in this structure results in an increased potential for a hydrogen bond contact with residue Q40 (in yellow) of CD4, since the distance is reduced from 3.7 to 2.4Å (Figure 8B), while the maximum distance between a donor-acceptor pair to form a hydrogen bond is usually considered to be 3.3Å, approximately. We modeled the structure of DS17 based on the crystal of HIV-1_JR-FL _gp120, and found that both wild-type N283 and mutant T283 appeared to be at a distance from Q40 that suggests that the formation of a hydrogen bond contact is unlikely (Figures 8C and 8D). Unfortunately, the BR gp120 could not be modeled with any of the reported gp120 crystal structures, while the SPL gp120 did return a good model based on the HIV-1_YU-2 _gp120 crystal structure (1G9N) [10]. This model suggested that both wild-type T283 and mutant N283 would be at a distance from Q40 that would allow the formation of a hydrogen bond (Figures 8E and 8F), although it would be more likely with N283 than with T283 since the distance is notably reduced. However, as reported above, we did not find any difference in CD4 dependence or macrophage tropism between the wild-type and mutant BR, SPL and DS17 Env, therefore confirming that the role of N283 in these phenotypes is highly context-dependent.

Modeling studies are always to some extent speculative since they are based on previous crystal structure data and sequence information. However, we evaluated the reliability of these molecular models using Verify3D , which determines the goodness of a model based on an available structure [96-99]. This analysis has confirmed the goodness of our models for DS17 and SPL Env, since for both we obtained scores greater than zero throughout gp120 on the Verify3D plot. Regions of bad fitting and loop regions tend to score zero or close to zero, and in fact a large proportion of BR gp120 (corresponding to the structured inner domain), which we could not model, had values close to zero with the published gp120 crystal structures. Therefore, we are confident with the models presented above.

## Conclusion

We had previously found that brain-derived Env from an individual with HIV-1 encephalitis have lower CD4 but not CCR5 dependence, increased fusogenicity and reduced sensitivity to inhibition by anti-CD4 mAbs and the fusion inhibitor T-1249, but not by the CCR5 antagonist TAK-779, than their splenic counterparts [54]. We hypothesized that the brain gp120 would contain the determinants for low CD4 dependence and higher avidity for CD4, while gp41 could likely play a role in the increased fusogenicity and reduced sensitivity to fusion inhibitors. In the present study, we have used chimeric and mutant Env and have identified that the V1-V3 region of the brain gp120 contains the determinants not only for low CD4 dependence, but also for the increased fusogenicity, enhanced macrophage tropism and reduced sensitivity to fusion inhibitors and the small molecule BMS-378806. Macrophage tropism had already been linked to low CD4 dependence and increased fusogenicity [31,39,41,53,55,57,67] and our study identified the specific gp120 region from a brain-derived, CCR5-using, macrophage-tropic Env conferring these phenotypes. The chimera containing the V1/V2 region of the brain-derived Env in the context of the spleen Env displayed an intermediate phenotype with reduced low CD4 dependence, macrophage tropism and fusogenicity, as compared to BR, but still different from SPL. This confirms that brain gp120's V3 region plays a role in the macrophage tropism of this Env, but also suggests that it is the V1/V2 region the main determinant for these phenotypes. However, regarding the sensitivity to entry inhibitors, although there is a clear relationship between macrophage tropism and diminished sensitivity to BMS-378806 (similarly to Peters et al. [56]), this is not the case with HNG-105, since some macrophage-tropic Env (Bv1v3, Bv1v2, BaL) show reduced sensitivity similar to the non-macrophage tropic Env SPL and SB, and quite different from the other macrophage-tropic Env, BR, BR(N283T), BS, DS17 and DS17(N283T). This could relate to the proposed allosteric mechanism of action of HNG-105.

Potentially, differences in the level of expression between wild-type, chimeric and mutant Env could have an impact on the phenotypes observed; however, it is not likely that this is the case since luciferase activity in cell-to-cell fusion assays was always between 100 and 1000-fold higher than the background levels, and pseudotype infection of HOS-CD4-CCR5 cells showed limited variability between pseudotype stocks but always remained at least 1000-fold higher than background levels. In addition, the fact that many pseudotypes display an opposite sensitivity to HNG-105 than to anti-CD4 mAb, BMS-378806 and fusion inhibitors supports the specificity of the results.

Diminished sensitivity to co-receptor and fusion inhibitors has been shown to correlate with higher affinity of gp120 for the co-receptor molecule CCR5 and with faster fusion kinetics [100,101]. In this set of Env, reduced sensitivity to T-1249 appears to correlate with lower CD4 dependence, increased fusogenicity and enhanced macrophage tropism, but not with a difference in the interaction with CCR5. Resistance to fusion inhibitors is primarily acquired both *in vitro *and *in vivo *through mutations in HR1 and the association between reduced sensitivity and mutations in HR2 has rarely been reported, and always in the presence of additional mutations within HR1 [102,103]. While there were no amino acid differences in the HR1 region of gp41e between brain and spleen clones, HR2 contained several amino acid changes which involved residues that can be considered polymorphic (moderately to highly variable). Thus, it was possible that the conserved amino acid sequence in the HR2 region of brain Env could result in an alteration/improvement of the interaction between HR2 and HR1, potentially increasing the fusogenicity of brain-derived Env and making the fusion process more resistant to the action of fusion inhibitors. This could be of great relevance since it might relate to the frequent finding of multinucleated giant cells in the CNS (while they are absent in most other tissues *in vivo*) and to the potential role of Env-mediated cell-to-cell fusion of infected and uninfected cells in the development of HIV encephalitis and the onset of neurodegeneration. Our results however demonstrate that, at least in these Env backgrounds, the determinants for increased fusogenicity and reduced sensitivity to fusion inhibitors lie in gp120 and overlap with those conferring low CD4 dependence, increased avidity for CD4 and enhanced macrophage tropism. It is possible that the genetic changes resulting in an altered Env conformation that confers lower CD4 dependence, higher avidity for CD4 and increased fusogenicity, may also affect the interaction with certain, but not all, entry inhibitors, with the difference potentially being on changes on the accessibility of the key residues that interact with those inhibitors due to the conformational alterations.

## Competing interests

The authors declare that they have no competing interests.

## Authors' contributions

FR, BQ and MN performed molecular and cell biology work, cell-to-cell fusion and viral infectivity and inhibition assays, as well as preliminary analysis of the data. SC performed biosensor experiments and modeling, and contributed discussion throughout the study. SNM contributed important planning and discussion on the experiments performed throughout the project. JMG conceived and designed the study, analyzed data and wrote the manuscript. All authors read and approved the final manuscript.
